# Multi-cohort cerebrospinal fluid proteomics identifies robust molecular signatures for asymptomatic and symptomatic Alzheimer’s disease.

**DOI:** 10.21203/rs.3.rs-3631708/v1

**Published:** 2024-02-16

**Authors:** Carlos Cruchaga, Muhammad Ali, Yuanyuan Shen, Anh Do, Lihua Wang, Daniel Western, Menghan Liu, Aleksandra Beric, John Budde, Jen Gentsch, Suzanne Schindler, John Morris, David Holtzman, Maria Fernández, Agustín Ruiz, Ignacio Alvarez, Miquel Aguilar, Pau Pastor, Jarod Rutledge, Hamilton Oh, Edward Wilson, Yann Le Guen, Rana Khalid, Chloe Robins, David Pulford, Laura Ibanez, Tony Wyss-Coray, Yun Ju Sung

**Affiliations:** Washington University School of Medicine; Washington University School of Medicine; Washington University School of Medicine; Washington University School of Medicine; Washington University School of Medicine; Department of Psychiatry, Washington University School of Medicine, St. Louis, MO, USA; Washington University in St. Louis; Washington University School of Medicine; Washington University School of Medicine; Washington University; Knight Alzheimer Disease Research Center; Washington University in St. Louis; Washington University School of Medicine; Ace Alzheimer Center Barcelona; Fundació Docència i Recerca MútuaTerrassa, Terrassa, Barcelona, Spain; University Hospital Mutua Terrassa; University Hospital Germans Trias i Pujol; Stanford University; Stanford University; Stanford University; Stanford University; Stanford; GlaxoSmithKline; GlaxoSmithKline; Washington University in St. Louis; Stanford University; Washington University Medical School

## Abstract

Changes in Amyloid-β (A), hyperphosphorylated Tau (T) in brain and cerebrospinal fluid (CSF) precedes AD symptoms, making CSF proteome a potential avenue to understand the pathophysiology and facilitate reliable diagnostics and therapies. Using the AT framework and a three-stage study design (discovery, replication, and meta-analysis), we identified 2,173 proteins dysregulated in AD, that were further validated in a third totally independent cohort. Machine learning was implemented to create and validate highly accurate and replicable (AUC>0.90) models that predict AD biomarker positivity and clinical status. These models can also identify people that will convert to AD and those AD cases with faster progression. The associated proteins cluster in four different protein pseudo-trajectories groups spanning the AD continuum and were enrichment in specific pathways including neuronal death, apoptosis and tau phosphorylation (early stages), microglia dysregulation and endolysosomal dysfuncton(mid-stages), brain plasticity and longevity (mid-stages) and late microglia-neuron crosstalk (late stages).

Alzheimer's disease (AD) is the most common age-related neurodegenerative dementia characterized by the presence of β-amyloid (Aβ) plaques and neurofibrillary tangles in the brain. AD remains an important threat to the aging population without any effective disease-modifying therapies available to date. The classification of AD proposed by the National Institute on Aging and Alzheimer's Association (NIA-AA) relies on biomarkers of amyloid (A), tau (T), and neurodegeneration (N) which constitute the ATN framework.^1^ Despite their diagnostic utility, these markers only capture a fraction of the intricate pathophysiology of AD. Genetics has also substantially advanced our understanding of AD heritable risk, revealing the complex polygenic nature of this disorder with an estimated genetic heritability between 58% to 79%.^2^ However, the interplay between AT(N) changes and the precise influence of genetic risk factors on the biological pathways underlying AD pathophysiology is not always clear.^3^

To gain comprehensive insights into the biological implications of AD, further analysis utilizing complementary -omics methodologies is often necessary. To this end, transcriptomic profiling has emerged as a widely employed approach to quantify mRNA transcripts in the post-mortem AD brains.^4^ The resulting transcriptomic data have also been integrated with AD genetic risk information to understand disease pathophysiology.^5^ Nevertheless, the proteins and metabolic pathways they regulate are frequently cited as the ultimate biological effectors of both genetic and environmental risk factors in AD. Therefore, high-throughput omics-based investigations in biological fluids such as cerebrospinal fluid (CSF) and plasma are needed to further gain mechanistic insights into the molecular processes involved in AD pathogenesis and prioritize connections to relevant clinical and neuropathological traits.

CSF serves as a valuable source for understanding different biochemical changes occurring in the brain during neurodegenerative disorders, offering insights into their underlying pathobiology.^6^ The classical AD CSF biomarkers include Aβ_42_ or its ratio (Aβ_42/40_), hyperphosphorylated tau (pTau) or total tau (tTau), and neurofilament light chain (NFL), which indicate the senile plaque pathology, formation of neurofibrillary tangles, and axonal degeneration in the brains, respectively.^7^ Alterations in the protein levels of these biomarkers, among others, can be detected years before the symptoms of AD appear.^8,9^ While these established pathological markers are widely employed for early AD diagnosis in research and clinical settings,^10^ they have limited utility in capturing the biological diversity of AD.^11–13^ Therefore, a systematic exploration of the CSF proteome holds the potential for identifying novel markers that reflect the multifaceted pathophysiology of AD. Besides refining the biological definition of AD, it can also provide crucial insights for developing robust AD prediction models that are independent of Aβ and tau pathology.

A growing body of literature,^14–18^, including our own,^19^ has leveraged proteomics datasets from CSF and plasma for identifying several pathways in AD including innate immune response and inflammation, oxidative stress, energy metabolism, and mitochondrial function. While the existing proteomics approaches have contributed significantly, they have been relatively limited in their coverage of target analytes that range between 453 to 4,001 protein analytes profiled using mass spectrometry^17,14^. This limited detection power of the existing approaches is primarily due to the extraordinary complexity and broad dynamic range of protein concentrations in the CSF and plasma.^20^ Furthermore, the limited sample of these studies, with samples sizes lower than 1000 samples,^16^ is also a significant hurdle in deriving statistically significant findings. Altogether, the utilization of low throughput protein profiling techniques and the limited sample size of the existing studies have significantly hampered their potential for identifying additional biomarker signatures and providing novel candidates that can serve as effective disease-modifying targets.

In order to identify significant alterations in the AD CSF proteome, create robust prediction models, and identify functional pathways compromised in AD, we have generated and analyzed high-throughput proteomics data from 2,286 participants in a three-stage study (Fig. 1). Then, the proteins associated with AD were used to create prediction models and for pathway and cell-type enrichment analyses in order to determine pathways implicated on AD pathogenesis.

## Results

### Study design

In this study, we used SomaLogic Somascan assay for measuring the protein levels of 7,029 analytes in CSF of 2,286 participants from Knight ADRC,^21^ FACE, ADNI, and Barcelona-1 cohorts. We employed a three-stage analytical approach (stage 1, stage 2, and meta-analysis) to identify robust proteomic alterations in the AD CSF proteome. Based on an AT(N) paradigm, the discovery analysis (stage 1) was performed in the Knight ADRC and FACE cohorts (n=1,170; A^−^T^−^ = 680, which correspond to biomarker negative individuals, and A^+^T^+^ = 490, or biomarker positive individuals). The significant proteins after false discovery rate correction (FDR < 0.05) were further replicated in stage 2 using ADNI and Barcelona-1 cohorts (n=593; A^−^T^−^ = 235 and A^+^T^+^ = 358). Finally, a meta-analysis of stage 1 and 2 was performed to identify robust proteins associations passing a more stringent Bonferroni correction (Bonf < 0.05) criteria. We further validated these proteins in a completely independent CSF proteomics cohort (Stanford ADRC; A^−^T^−^ = 80 and A^+^T^+^ = 27) profiled using a different protein quantification platform (Somascan 5K).

Using lasso regression, we identified a distinctive signature of 11 proteins with robust and high predictive power for AD (AUC = 0.97–0.99) in three independent cohorts (stage 1, stage 2, and validation). The identified proteomic signature was unique to AD and showed no to very low predictive power for other dementia such as frontotemporal dementia (FTD; AUC = 0.61), dementia Lewy body (DLB; AUC = 0.73), or Parkinson’s disease (PD; AUC = 0.57). The developed proteomic signature displayed significant association with disease progression (β = 0.35, p = 2.1×10^−04^) and individual’s probability of not developing AD (p = 2.2×10^−58^).

**Figure 1:**
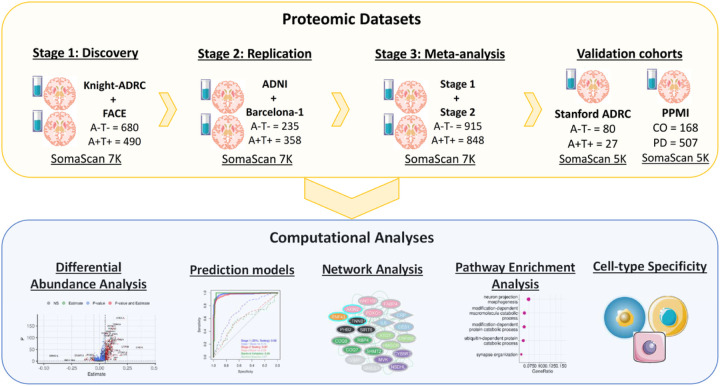
Schematic of experimental and analytical workflow. A three-stage (Stage 1, Stage 2, Meta-analysis) analytical workflow was used to identify significant changes in the AD CSF proteome (A^−^T^−^ vs. A^+^T^+^). The proteomic signatured identified in the meta-analyses was further tested in an external validation cohort (Stanford ADRC). The identified proteomic changes were subsequently used for creating robust disease prediction models, delineating the protein abundance trajectory across different AD stages (A^−^T^−^, A^+^T^−^, A^+^T^+^), conducting pathway and cell type enrichment analysis, as well as generating protein-protein interaction networks to understand AD biology.

A comprehensive examination of protein abundance across various AT groups (A^−^T^−^, A^+^T^−^, and A^+^T^+^) revealed distinct protein pseudo-trajectories (estimating protein longitudinal trajectories based on cross-sectional data) that span the entire AD continuum. Based on these disease stage- we obtained four different group of proteins, with unique pseud-trajectories. Group-specific pathway enrichment was performed to understand biological processes compromised during different stages of AD continuum. Each group displayed enrichment for several biological systems (nervous system, immune response, biosynthesis, and signal transduction) and specific brain cell types (neuron, astrocytes, and microglial cells). Overall, the disease and pathway enrichment analyses highlighted several neurological disorders (e.g., AD, tauopathy, and synucleinopathy) and neuronal functions (neuron projection morphogenesis, synapse assembly, and axonogenesis) to be significantly enriched (FDR < 0.05) in the altered AD CSF proteome (Table 1, Fig. 1).

### Identification of AD-specific CSF proteomic alterations

**Figure 2:**
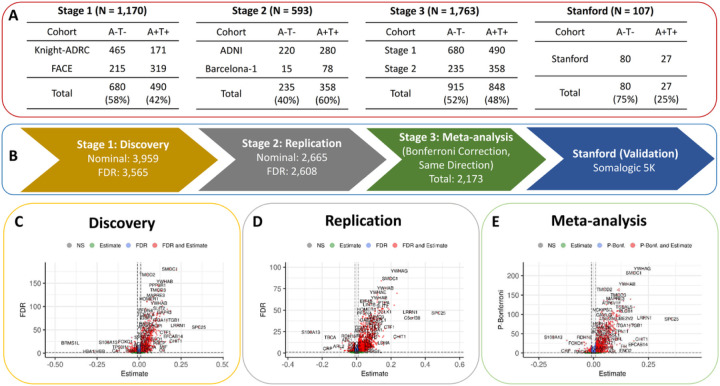
Differential abundance analysis of AD CSF proteomics. A) We used AT(N) framework to identify proteins displaying a significant association between A^−^T^−^ and A^+^T^+^ individuals in the stage 1 (n=1,170) and stage 2 (n=593). The results from both these stages were further meta-analysed (stage 3) to obtain a final set of proteins showing consistent associations across all stages. B) A three-stage study design (discovery, replication, meta-analysis) was employed to identify AD-specific proteomics alterations in the CSF. The robustness of the meta-analysis results was further validated in an independent study (Stanford ADRC; n=105). C) Volcano plots displaying proteins with significantly increased and decreased abundance in the A^+^T^+^ individuals in comparison to A^−^T^−^. The dotted lines on the x- and y-axis of the volcano plot indicate the thresholds for estimate (0) and FDR (0.05), respectively, except in the case of meta-analysis where Bonferroni correction was applied. Proteins on the right side of the dotted lines indicate higher abundance in A^+^T^+^ in comparison to A^−^T^−^ individuals, whereas, the ones on left side indicates lower abundance.

We performed a three-stage study to identify significant alterations in AD CSF proteome (Fig. 2B). In the first stage, a discovery analysis was performed on 1,170 individuals (A^+^T^+^ = 490, A^−^T^−^ = 68) from the Knight ADRC and FACE studies (Fig. 2A). We identified 3,565 with significantly different levels (FDR < 0.05) between A^−^T^−^ (biomarker negative and a proxy for controls) and A^+^T^+^ (biomarker positive and a proxy for AD cases) individuals (Fig. 2C and Supplementary Table 1). Consistent with previous proteomic studies, some of the significantly up-regulated proteins included known AD biomarkers such as SMOC1 (FDR = 2.8×10^−181^), 14–3-3 protein YWHAG (P = 4.1×10^−179^), PPP3R1 (FDR = 1.8×10^−146^), and NRGN (FDR = 1.8×10^−90^). ^17–19,22–24^

In the second stage, the protein that showed significant associations in stage 1 were further tested in the stage 2 that comprises 593 individuals (A^−^T^−^ = 235, A^+^T^+^ = 358) from ADNI and Barcelona-1 (Fig. 2A). Of the 3,565 identified proteins in stage 1, 2,608 replicated in stage 2 after FDR and with consistent effect direction (Fig. 2D and Supplementary Table 2). Of these 1,693 were upregulated in A^+^T^+^ (cases) compared to A^−^T^−^ (controls), and 915 were downregulated.

In the third stage, we performed a meta-analysis to combine the p-values from stage 1 and 2, for those proteins that replicate in stage 2 and applied a stringent Bonferroni correction to minimize the chances for false-positive results (Fig. 2A). The meta-analysis resulted in 2,173 proteins associated with AT status after Bonferroni correction (Fig. 2E and Supplementary Table 3). Finally, we validated these findings by using CSF proteomics data from an independent study (Stanford ARDC) that employed a different proteomic panel (Somascan 5K). As this validation cohort had a limited size (n=132 and Table 1), we assessed the consistency of effect size and significance (p-value) across all these studies. We observed a strong correlation between the effect size (corr = 0.90, p = 3.3×10^−187^) and p-values (corr = 0.82, p = 1.5×10^−138^) of the meta-analysis and the Stanford ADRC study (Figure S2). This unbiased validation confirms the platform-independent robustness of our meta-analysis results. We considered the 2,173 proteins that passed Bonferroni correction in stage 3 for downstream analyses: disease prediction models, and pathway enrichment (Fig. 1).

### Identification of a robust and AD-specific prediction model

**Figure 3:**
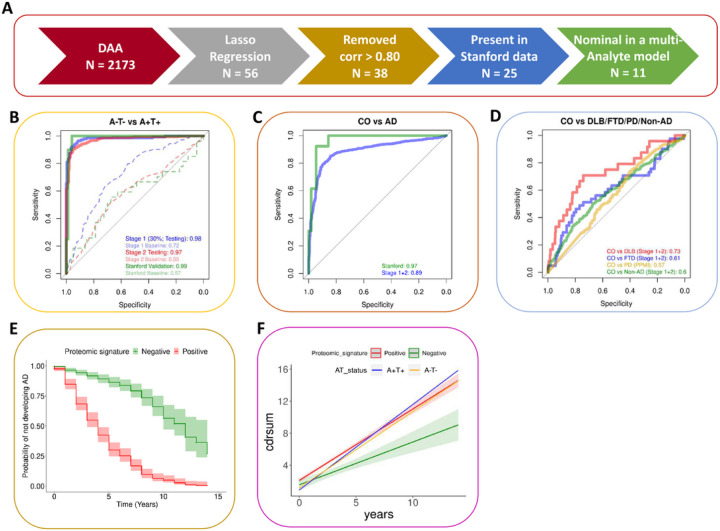
Performance of 11-protein AD prediction model. A) Derivation of 11 protein panel AD prediction model. B) Performance of identified AD prediction model in comparing A^−^T^−^ and A^+^T^+^ individuals across different discovery and replication datasets. C) Performance of identified AD prediction model when applied to classify individuals based on clinical diagnosis (AD = Alzheimer’s disease, CO = healthy controls). D) Predictive power of identified AD prediction model in case of other related dementias including dementia Lewy body (DLB), frontotemporal dementia (FTD), Parkinson’s disease (PD), and other non-AD individuals in comparison to healthy controls. E) Rate of dementia progression over time for individuals predicted as proteomic signature–positive (red) and –negative (green) using 11-protein AD-specific CSF proteomic panel. No significant difference was observed in the rate of dementia progression for A^+^T^+^ (blue) and A^−^T^−^ (orange) individuals. F) Time-to-event (developing AD) analysis of individuals predicted as proteomic signature–positive (green) and –negative (red). Upper and lower 95% confidence intervals for both these groups are represented by slightly transparent regions around the actual slopes in the Kaplan-Meier curve.

Since the entire set of differentially abundant analytes (DAA; n=2,173), identified using multi-stage metanalysis, is too large for developing a clinically meaningful proteomics panels for AD diagnosis and prognosis, we used machine learning approaches to identify the minimum number of proteins with high prediction power (Fig. 3A). We used least absolute shrinkage and selection operator (Lasso) regression model^25^ on 70% of the stage 1 (stage 1 training; n=819) for training. The Lasso regression model with five-fold cross-validation identified 56 proteins. Proteins displaying high correlation (Pearson correlation > 0.8) between the abundance levels in the stage 1 data were removed to further reduce the size of proteomic signature. Since the performance of identified proteomic signature was also assessed in an independent study (Stanford ADRC) that used a different protein quantification platform (Somascan 5K), only proteins overlapping between the proteomic signature and Stanford ADRC data (n=25) were kept. Finally, a set of 11 proteins, which significantly contributed to the prediction (P < 0.05 in the multi-variant model; Supplementary Table 4) were kept. The identified proteomic signature included some of the well-known AD-associated proteins such as YWHAG,^18,22^ PIN1,^26^ and EZR.^27^

This model (11 proteins and specific weights) was assessed in the stage 1 testing (30% of stage 1 data; n=351), stage 2 (replication; n=593), and external validation (Stanford ADRC; n=107) datasets. This model showed strong prediction power for classifying A^+^T^+^ vs A^−^T^−^ individuals, with an area under the curve (AUC) of 0.98 and 0.97 for stage 1 testing and stage 2 datasets respectively, and 0.99 in the independent Stanford ADRC cohort (Fig. 3B). Positive predictive value (PPV) and negative predictive value (NPV) were >0.86 in all cases (Supplementary Table 5). The performance of the baseline model, which only used age and sex for predicting AT status was significantly low for stage1 testing, stage 2, and Stanford validation cohorts, with an AUC of 0.72, 0.59, and 0.57, respectively (Fig. 3B).

We also analyzed if the same model can predict clinical diagnosis (Controls = 724, AD = 882), and obtained an AUC of 0.89 for stage 1+2, and 0.97 and Stanford ADRC (Fig. 3C). These high AUC suggests the robustness of our prediction model in stratifying clinical AD individuals from controls, as well as AT biomarker status.

To further assess the specificity of this prediction model for AD, we also applied it (same proteins, weights and cut-off as identified in Stage-1 training) to other dementia disorders including dementia Lewy body (DLB; n=25), frontotemporal dementia (FTD; n=42), and Parkinson’s disease (PD; n=507), as well as other non-AD individuals (n=335) and healthy controls (n=1,157). We observed that model did not have a strong prediction power for these non-AD dementias and PD, with AUC ranging from a maximum of 0.70 in the case of DLB to a minimum of 0.44 for PD (Fig. 3D). Overall, these results suggest that we have identified a unique signature of 11 proteins that showed consistently high prediction power for predicting AD clinical or biomarker status. This identified proteomic signature is specific to AD as it showed very low power for other dementia such as FTD, DLB, or PD.

### Assessing progression to dementia and rate of memory decline

Next, we asked if the identified CSF 11-proteins signature can reliably distinguish between slow and fast progressors. For this analysis, we focused on individuals with an AD-diagnosis at lumbar puncture and rate of memory decline was modeled using change in Clinical Dementia Rating sum of boxes (CDR-SB) per year. We observed a significant separation between the regression slopes for individuals predicted as proteomic signature-positive and -negative (Fig. 3E; red and green slopes, respectively). Individuals positive for the proteomic signature presented faster rate of progression (β = 0.35, p = 2.1×10^−04^). No difference between the slopes was observed between A^−^T^−^ vs A^+^T^+^ individuals (Fig. 3E; blue and orange slopes).

We also performed a time-to-event analysis to assess if our proteomic signature can also determine if cognitive normal individuals at lumbar puncture are more likely to develop AD. We observed that individuals positive for the 11-protein panel displayed a significantly high probability of developing AD (p = 2.2×10^−58^) in comparison to individuals that were negative for the proteomic signature (Fig. 3F). In particular, the individuals positive for the 11-protein panel displayed almost 100% of the individuals develop AD in the 10-year interval post-first clinical assessment, whereas the individuals negative for this panel showed 35% probability of developing AD in the same time span.

In summary, these results indicate that the identified AD CSF proteomic signature is a better predictor of dementia progression than the known AT status. Furthermore, individuals that are predicted to be positive for this proteomic signature exhibit significantly low probability of not developing AD as compared to their counterparts that are negative for this signature.

### CSF proteome exhibit distinct protein expression patterns throughout the AD continuum

Following the AT classification system, we categorized individuals into three groups: biomarker negative individuals (A^−^T^−^), individuals in early AD stages: amyloid positivity but tau negativity (A^+^T^−^), and full biomarkers positive individuals (A^+^T^+^), which cover the entire AD continuum. The goal of this analyses is to determine how the protein levels change across the AD continuum (pseudo-trajectories), determine if there are specific patterns of those changes and the pathways associated with those changes.

**Figure 4:**
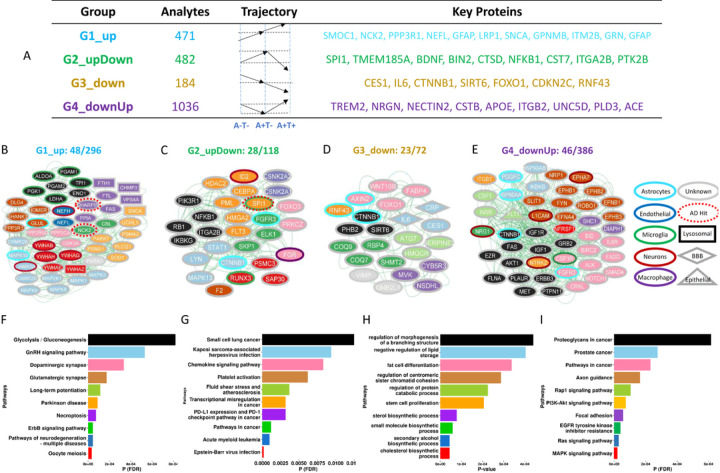
Pathway enrichment and network analyses identify well established and novel proteins and pathways implicated in AD. A) Grouping of differentially abundant proteins based on their distinct alteration trajectories in the AD continuum. B-E) Protein-protein interaction (PPI) networks were obtained using STRING database for proteins constituting the top 10 functional pathways (F-I) enriched in individual protein groups.

Based on the differences in the estimates and their significance across three independent differential abundance analyses (A^−^T^−^ vs. A^+^T^−^, A^+^T^−^ vs. A^+^T^+^, and A^−^T^−^ vs. A^+^T^+^), we identified four distinct groups of proteins (Fig. 4A and Supplementary Table 6). Specifically, group one (G1) included 471 proteins that showed consistently “linear increase” in protein abundance from healthy controls (A^−^T^−^) to asymptomatic (A^+^T^−^) to AD (A^+^T^+^) stage. The second group (G2) included a set of 482 proteins that followed an “up-down” trend, i.e., they showed an increase in protein abundance from biomarker negative to early stages and then a decrease to full biomarker positive. Group 3 (G3) included a set of 184 protein analytes that showed a consistent “linear decrease” from biomarker negative to positive. Finally, group four (G4) showed the exact opposite behavior of G2, a “down-up” trajectory, where an initial decrease was followed by an increase.

The G1 (linear increase) includes key AD-associated proteins such as SPARC-related modular calcium-binding protein 1 (SMOC1,^28^ Extended Data Fig. 1), Neurofilament Light Chain (NEFL)^29^, Glial Fibrillary Acidic Protein (GFAP)^30^, Granulin Precursor (GRN)^31^, Protein Phosphatase 3 Regulatory Subunit B, Alpha (PPP3R1)^32^, and Alpha-Synuclein (SNCA)^33^. Besides having these established AD biomarkers, this group also included NCK Adaptor Protein 2 (NCK2) and SHANK Associated RH Domain Interactor (SHARPIN) which are located on two known AD risk loci.^34^ Recent studies have revealed that SMOC1 protein in the brain colocalizes with Aβ plaques^35^ and its CSF levels increase almost 30 years before AD symptom onset.^36^

The G2 (up-down) also includes proteins located on multiple known AD-risk loci such as SPI1 and Protein Tyrosine Kinase 2 Beta (PTK2B), as well as other proteins known to be implicated on AD or neurodegeneration such as Brain-Derived Neurotrophic Factor (BDNF)^37,38^, Cathepsin D (CTSD)^39,40^, and Nuclear Factor Kappa B Subunit 1 (NFKB1) ^41,42^. Some of the key proteins contained in the G3 (linear decrease) group include Carboxylesterase 1 (CES1)^43^, Interleukin 6 (IL6)^44,45^, and Forkhead Box O1 (FOXO1)^46,47^, which have been implicated in various metabolic, age-, and immune system-related mechanisms that underlie AD pathogenesis. Finally, and consistent with previous study,^48^ we found Triggering Receptor Expressed On Myeloid Cells 2 (TREM2) in the G4 (down-up), which showed a decrease from controls to the asymptomatic stage but then significantly elevated levels are noticed in AD individuals. Besides TREM2, G4 contains various other proteins that have been implicated in AD, including Apolipoprotein E (APOE)^49^, Neurogranin (NRGN)^50,51^, ADAM Metallopeptidase Domain 17 (ADAM17)^52,53^, and Nectin Cell Adhesion Molecule 2 (NECTIN2)^54^. Overall, these results identified four groups of proteins based on their estimated trajectories based on the AD continuum, with each group including known proteins implicated on AD or neurodegeneration.

### Network and pathway analysis of the CSF proteome reveal novel proteins related to AD pathophysiology

In order to identify the specific biological process that each of those groups, with unique trajectories, we conducted a functional pathway enrichment analysis using a set of selected topologically important proteins (Fig. 4F-I). To further gain a systems-level understanding of the proteins part of specific pathways, we utilized STRING database^55^ and extracted protein-protein interaction (PPI) information between the constituent proteins from the top 10 pathways.

G1 captures neuronal death, apoptosis and defects in phosphorylation/dephosphorylation. Specifically, proteins in G1 were enriched in the nervous system related pathways (Fig. 4F and Supplementary Table 7) including *pathways of neurodegeneration – multiple diseases* (FDR = 1.6×10^−05^), *glutamatergic* (FDR = 1.8×10^−04^) or *dopaminergic synapse* (FDR = 3.40×10^−04^) and *Parkinson’s disease* (FDR = 9.2×10^−05^) among others. The dopaminergic synapse pathway includes known kinases (GSK3A), and phosphatases (PPP3CA and PPP2R5D). GSK3A and calcineurin (PPP3CA) are known to be involved on tau phosphorylation regulation^56^ and PPP2R5D is known to cause an autosomal dominant neurodevelopmental disorder, Jordan’s syndrome,^57^ although this is the first time this protein is implicated on AD. The glutamaergic pathway includes proteins known to be part of the causal AD pathways such as another proteoforms of calcineurin (PPP3R1) reported to be associated with phosphor-tau levels and rate of memory decline,^56^ or HOMER1.^58^. This pathway also includes DLG4 and GLUL, both neuronal-specific proteins, involved on signal transduction. The G1 group also includes several proteins implicated on Parkinson such as PRKN, SNCA and PARK7^59,60^. The identification of these proteins could explain why around 30% of the AD cases have Lewy Body pathology, which is normally found in PD. The G1 network also contained NCK2 and SHARPIN, two previously known AD risk loci,^34^ associated with the *ErbB signaling pathway* (FDR = 4.65×10^−05^), and *Nectoproptsis* (FDR = 7.52×10^−05^), respectively. The nectoproptsis pathway also include other proteins such as SHARPIN what we recently found to be genetically dysregulated in AD cases and to be part of the causal pathways by performing pQTL mapping couple with colocalization and Mendelian Randomization analyses. All these results suggesting that some of these proteins could not only be pure biomarkers but also part of causal pathway of AD. On the other hand, some known biomarkers included in this group includes both NEFL and NEFH.^61,62^ We also found multiple 14–3-3 proteins (e.g. YWHAB, YWHAG, and YWHAH) to be part of this group extending our previous results,^63^ which are predicted to be neuronal specific and are part of the cell division pathway (FDR = 5.29×10^−08^). Multiple recent studies suggest that mosaic mutations resulting from mitosis defect^64^ could also be involved in AD pathogenesis.

In contrast of G1 (lineal increase) which seems to capture early neuronal death, the G2 (down-up) group is capturing immune response glia-specific and endolysosome pathways, including *platelet activation* (FDR = 0.006), *chemokine signaling pathways* (FDR = 0.008), and *acute myeloid leukemia* (FDR = 0.001; Fig. 4G and Supplementary Table 8), which likely as a response to early neuronal death. SPI1, a microglial marker gene located on a well-known AD risk locus,^34^ was be a part of transcriptional misregulation in cancer pathway, most likely regulating the microglial inflammatory response in AD^65^. We observed a consistently low abundance levels of SPI1 in the CSF of A^+^T^+^ individuals compared to A^−^T^−^ in both stage 1 (estimate = −0.001, FDR = 2.4×10^−07^) and stage 2 (estimate = −0.01, FDR = 1.5×10^−04^). In line with our findings, a decreased level of SPI1 in both primary human microglia and the BV-2 mouse microglia cell line has been shown to be associated with reduced phagocytic capacity of the cells^65–67^, further supporting these findings. Other proteins of this pathway that interact with SP1 include FLT3 which is important for the normal development and the immune system and is a drug target for acute myeloid leukemia (AML).^68^. PML, another protein identified in our analyses is also part of this group, interacts with SP1 and is a tumor suppressor protein that is associated with acute promyelocytic leukemia.^69^ CEBPA, another protein involved in leukemia^70^ is also part of this network. Some other important proteins included in the G2 group included signal transducer and activator of transcription-1 (STAT1), NFKB1, and Forkhead Box O3 (FOXO3) that have previously been shown to be linked with the inflammatory response in AD brains.^71–73^. In summary the G2 group is able to capture many novel proteins that are part of the inflammatory and immune response pathway that may become dysregulated due to early neuronal death and apoptosis.

The G3, which displayed linear decrease throughout the AD continuum, seems to be capturing proteins and pathways related to brain plasticity or mechanism trying to compensate for AD-related pathology, including pathways part of biosynthesis-related biological processes (Fig. 4H and Supplementary Table 9), such as *cholesterol biosynthetic process* (P = 4.4×10^−05^), *sterol biosynthetic process* (P = 7.8×10^−05^), and *stem cells proliferation* (P = 2.1×10^−04^; Fig. 4D). Numerous proteins within this group include AXIN2 and CTNNB1.^74^ AXIN2 is a suppressor of Wnt/β-catenin signaling known to affect mitochondrial biogenesis, which is linked to several neurodegenerative disorders, including AD.^75^ Consistent with our findings, a significant reduction (~70%, P < 0.001) in soluble β-catenin (CTNNB1) levels has already been shown in AD brains as compared to controls^76^, and accumulation of this protein is a marker of ubiquitination and rapid proteasomal degradation.^77^ Part of the same pathway as CTNNB1, is SIRT6 which several studies that higher SIRT6 levels area associated with longer lifespan^78,79^, which is in line with our findings as we found these proteins decreased in AD cases.

Lastly, proteins within G4 group (down-up) captures a difference microglia activity to that of G2 (up-down; Fig. 4E), as it has the opposite pseudodirectory pattern, and it also captures cell-to-cell crosstalk. Proteins in this group were enriched in in the *MAPK signaling* (FDR = 3.7×10^−07^), *Ras signaling* (FDR = 5.4×10^−06^), *Rap1 signaling* (FDR = 1.1×10^−04^), and different cancer–related pathways e.g., pathways in cancer (FDR = 2.4×10^−04^) and prostate cancer pathways (FDR = 2.8×10^−04^; Fig. 4I and Supplementary Table 10). Some of the important highlights of this network include CSF1, and CSF1R, involved in several signally pathways, and key proteins to maintain proper microglia activity.^80–82^. Other microglia and inflammation related proteins include, FAS, IGF1, and IGF1R proteins, which have been implicated in the pathogenesis of AD and other amyloidosis disorders^83^. Other key proteins in this group that were not part of the top 10 pathways included TREM2, APOE, PLD3, and NRGN, which have already been implicated in AD, known to be involved on microglia or lysosome activity.^49,50,84,85^. At the same time, we observed multiple proteins in this group to be the protein-encoding marker genes for neurons (NRG1, a co-receptor for of RENL, a recent gene identified in AD resilience,^86^, NTRK2, L1CAM, and EPHA7) and astrocytes (CTNNB1, FGFR3, and PDGFC), with most of them being enriched in signaling related processes which suggest microglia neuron communication.

In summary, by first grouping the proteins based on their trajectory and performing pathway analyses we have been able to identify specific mechanism affecting AD pathogenesis at different stages of the disease that other general pathways analyses would have missed (table ST11-ST15, extended results).

## Discussion

Cerebrospinal fluid (CSF) serves as a protective barrier for the central nervous system (CNS) and analyzing CSF proteome can contribute to the diagnosis of various CNS-related diseases, but our understanding of robust AD-specific CSF proteome alterations is currently limited.^87,88^ While numerous CSF proteomics investigations have focused on AD,^14–19,22,23^ none have examined a cohort of this magnitude (7,029 proteins in 2,286 individuals), thus hindering their ability to identify consistent proteomic changes and construct a reliable predictive model. Although previous studies have identified several novel protein markers for AD, most of which were also replicated in our analyses, their major limitations were the limited coverage of the proteome and relatively small sample size. Moreover, relatively fewer studies have covered the entire AD continuum,^14–18^ as many of them omitted the asymptomatic (A^+^T^−^) or mild cognitive impairment (MCI) stage, which is crucial for identifying early biomarkers for AD. In-depth CSF proteomic profiling of AD patients and controls has the potential to uncover disease-specific proteomic alterations, provide insights into the underlying biological processes, and translate these multifaceted findings into practical disease prediction models for better and early diagnosis. In this study, we generated and analyzed one of the largest AD CSF proteomic profiles from four independent cohorts, comprising 2,286 individuals, measuring 7,029 protein analytes.

Utilizing the AT(N) framework and a three-stage analytical approach (discovery (Stage 1), replication (Stage 2), and meta-analysis (stage 3)), along with rigorous multiple test correction (Bonferroni correction), we identified 2,173 proteins showing significant association between AD (A^+^T^+^) and controls (A^−^T^−^; Fig 2B,E). We observed that more than 97% of the significant proteins in Stage 1 also had the same direction in two different replication cohorts, highlighting the robustness of our analyses. Furthermore, a strong Pearson correlation was observed between the effect sizes (corr = 0.90, p = 3.3×10^−187^) and p-values (corr = 0.82, p = 1.5×10^−138^), when comparing the meta-analysis results (stage 3) against a completely independent cohort (Stanford ADRC; Extended Data Fig. 2), even though this study used a different platform (Somascan 5K instead of the 7K panel we used). This observation further validates the robustness of our findings. Using this approach, we verified previous findings on multiple proteins associated with AD dementia, detected using the same or different analytical techniques. For instance, proteins like SMOC1,^18,19,22^ YWHAG,^18,19,22^ ALDOA,^18,19,22^ ITGB2,^16,18,19,22^, SOD1,^16,18,19,22^ and NRGN^18,19,22^ showed significantly increased abundance in AD (A^+^T^+^), whereas, AHSG,^18,19^ CD74,^18,19^ and FLT3^18,22^ showed reduced levels in the CSF of AD individuals in comparison to healthy controls (A^−^T^−^).

Proteins displaying significant changes in the AD CSF proteome can be leveraged for creating prognostic biomarkers to identify individuals at high risk for disease. An existing study^16^ utilized CSF proteomic data from 425 individuals to propose an 8-proteins diagnostic panel with an AUC of 96% for distinguishing AD from controls in discovery and 0.94 (in replication; n=62), however this model also shoed high AUC for non-AD dementia was quite high= 0.80. Similarly, another study utilizing CSF proteomics data measured using 1.3K Somascan,^19^ introduced a 12 protein panel that distinguished sporadic AD from healthy controls with an AUC of 88% and 100% in discovery (n=717) and replication (n=110) datasets, respectively. Although both these approaches displayed reasonable model performance, they shared two important limitations. Firstly, they had a significantly low replication sample size (almost 6 times lower than discovery) resulting in less reliable AUC estimation.^89^ Secondly, a lack of systematic sensitivity and specificity assessment by testing the identified panels on other dementias (e.g., FTD, DLB, and PD). Our study addresses these limitations by considering a well-balanced sample size for discovery (stage 1) and replication (stage 2) cohorts as well as utilizing a completely independent validation cohort (Stanford ADRC), which used a different platform for proteomic quantification, for an unbiased assessment of biomarker performance across different cohorts. Within the large number of associated proteins, we identified and externally validated an 11-protein CSF AD proteomic signature capable of distinguishing patients with AD from cognitively healthy controls (with AUCs of 0.98 – 0.99; Fig. 3A and B), as well as from the asymptomatic individuals (with AUCs of 0.88 – 0.96; Extended Data Fig. 3). The identified biomarker is specific to AD, as its predictive power is significantly low (with AUCs of 0.44 – 0.70) when tested on other non-AD dementia datasets (e.g., FTD, DLB, and PD; Fig. 3D) further strengthening our hypothesis that the identified proteomic biomarker panel is AD-specific and a promising candidate for the development of clinical assays.

To further assess the diagnostic practicality of developed AD biomarker panel, we performed a rate of disease progression analysis and observed a significantly positive association (β = 0.35, p = 2.1×10^−04^) between biomarker positivity (A^+^T^+^) and faster progression for cognitive decline (Fig. 3E and Extended Data Fig. 4). We did not observe any significant effect of covariates like age and sex on this association (Extended Data Fig. 5). In contrast, when using the actual AT status, the regression model was not able to capture any difference (β = 0.10, p = 0.36) between the slow and fast progressors (Fig. 3E). These results suggest that identified CSF proteomic biomarker can reliably distinguish between slow and fast progressors, underscoring its promising potential in the clinical diagnostic settings. Although previous studies have applied this concept to explain the genetic architecture of the AD and its differential effect on different sexes^90,91^, where women displayed two-fold faster progression for cognitive decline than male, this avenue was not yet explored for assessing the predictive power of a disease-specific biomarker panel. To further complement these findings, we employed a time-to-event regression model^92^ and evaluated potential distinctions in probabilities for not developing AD between proteomic signature–positive and –negative individuals. Individuals positive for 11-protein signature exhibited a significantly lower probability of not developing AD (p = 2.2×10^−58^) when compared to individuals –negative for this signature (Fig. 3F). In particular, the individuals –positive for identified proteomic panel showed a disease conversion (incidence) rate of almost 100% in the 10-year follow-up from the first clinical assessment. In contrast, the individuals –negative for this panel displayed significantly lower (~35%) disease conversion rate in the same time span. In summary, these results highlight substantial variations in cognitive decline rate and survival time between proteomic signature–positive and –negative individuals, highlighting the potential of the identified AD proteomic panel for early disease detection.

The significantly altered CSF proteins span diverse mechanisms linked to AD pathogenesis, including several neuronal and immune system related functions as well as different neurological disorders, offering a new *in vivo* perspective on the complex nature of the disease (Fig. 4). Instead of performing pathway analyses for all associated proteins or based on protein correlations, using WGCNA-like approaches, we decided to apply a novel approach based on grouping the proteins based on their pseudo-trajectories along the AD continuum. This approach allowed us to disentangle novel biological pathways that otherwise could be eclipse in regular pathways analysis due among the large number of proteins associated with AD. Among the biological pathways significantly enriched in AD-specific proteomic alterations, in the first group (G1) of proteins based on their pseudo-trajectories includes pathways of neurodegeneration (FDR = 1.6×10^−05^) and tau phosphorylation, and apoptosis, which is likely capturing neuronal death (Supplementary Table 7–10 and Extended Data Fig. 6–9). The pathways of neurodegeneration include proteins related to Neurofilaments (NEFH and NEFL), among others. The gene NEFL is a putative biomarker of neurodegeneration^93^ and its corresponding protein level in plasma has been used for assessing cognitive decline and mild cognitive impairment in AD.^29^ NEFH is primarily associated with neurons, and elevated CSF levels of this protein have been detected across multiple neurodegenerative disorders^94,95^ along with AD^96^. The second group is capturing a unique set of microglia and immune-related proteins (SPI1 and RUNX3) involved on regulating neuroinflammatory response and displaying high transcriptomic expression in late-onset AD (LOAD),^97^ we also observed an elevation in their AD CSF levels. In addition, SPI1 has already been characterized as a known AD risk loci (Odds Ratio = 1.06, P = 5.3×10^−14^)^34^. This may be helpful for fully understanding how changes in brain microglia can contribute to the dysregulation of immune response in AD. The Rap1 signaling pathway regulates several cellular processes, including synaptic efficacy, cytosolic calcium influx, and neuronal repolarization.^98^ Dysregulation of these processes is among the earliest pathological events in both familial and sporadic AD,^99,100^ implying that devising interventions directed at this pathway could be notably effective when AD pathology becomes evident. The third group of proteins based on their pseudo-trajectory are enriched in proteins related to healthy aging/longevity and brain plasticity, likely capturing brain processes that are trying to compensate for the neuronal death and overall ongoing pathology due to disease. Some of the proteins in this group, could be targeted to delay or stop AD progression, although additional analyses will be needed. Finally, the last group also include many known microglia proteins (CSF1, CSF1R, TREM2), but this group showed opposite pseudo-trajectories to those microglia proteins in G2, suggesting that different microglia subpopulations and/or pathways play different roles on AD pathogenesis.

While this study analyzed a substantial number of 7,029 proteins and included 2,286 samples, it is not without limitations. Firstly, we observed 14% and 11% of the individuals clinically diagnosed as AD or controls to be biomarker–negative (A^−^T^−^) and –positive (A^+^T^+^), respectively, implying the potential influence of misdiagnosis. Secondly, we employed multiple external datasets for validation of our finding.

Some of these datasets used different proteomic profiling platforms (e.g., Stanford ADRC using 5K Somascan panel), leading to the omission of certain proteins identified during the discovery phase in the validation cohort. Although genomics and transcriptomics have contributed significantly to the development of clinical diagnostic assays,^4,101^ proteomics approaches have been relatively limited in their coverage of target analytes, primarily due to the extraordinary complexity and broad dynamic range of protein concentrations in the CSF or plasma.^20^ Lastly, since our study exclusively involves individuals from the non-Hispanic whites population, we cannot extend the assessment of the identified AD CSF proteomic biomarker to other racial groups, as demonstrated previously by Modeste et al.^22^

In summary, we have analyzed a large well-characterized AD CSF proteomics cohorts and identified novel proteins and pathways dysregulated in AD. Our study showed the potential of utilizing these proteomic alterations for developing robust and AD-specific biomarker panel with promising diagnostic applications in clinical assays. While further validation of this biomarker panel is warranted across different racial groups, we observed consistent and replicable results when tested in a completely independent cohort profiled using a different platform, underscoring the efficiency of the employed workflow for biomarker development. Overall, our findings display the potential of proteomic studies in advancing our understanding of AD biology and pathophysiology.

## Methods

### Study design

The aim of this study was to investigate AD CSF proteome alterations for identifying AD-specific proteomic signatures and examining the interactions between identified proteins to better understand the underlying AD biology. CSF samples used in this study were obtained from the Charles F. and Joanne Knight Alzheimer Disease Research Center (Knight ADRC, n=836),^21^ Alzheimer’s Disease Neuroimaging Initiative (ADNI, n=700), Fundació ACE Alzheimer Center Barcelona (FACE, n=618), and Barcelona-1 (n=132) cohorts (Table 1). Altogether, this constitutes one of the largest AD proteomic profiles, including 7,029 protein analytes measured in the CSF of a total 2,286 individuals, which were analyzed in a three-stage study. In stage 1, a discovery was performed in 1,170 samples from the Knight ADRC and FACE cohorts using the ATN framework (A^−^T^−^ = 680 and A^+^T^+^ = 490). In stage 2, the proteins that passed multiple test corrections (FDR < 0.05) in the stage 1 were further tested in 593 individuals (A^−^T^−^ = 235 and A^+^T^+^ = 358) from the ADNI and Barcelona-1 replication cohorts. In stage 3, we performed a meta-analysis encompassing both stages (1 and 2), and proteins demonstrating consistent effect sizes and surviving multiple Bonferroni corrections (Bonf < 0.05) were identified as significantly altered proteins in the AD CSF. The identified proteomic alterations further underwent a validation using completely independent CSF proteomic study (Stanford ADRC, n = 132) profiled with a different quantification platform. The identified CSF proteomic changes were utilized to develop robust AD-specific prediction models and categorize proteins into four different groups based on their varying trajectories across the AD continuum (A^−^T^−^, A^+^T^−^, A^+^T^+^). Besides assessing the performance of AD prediction model in three independents cohorts (stage 1, stage 2, Stanford ADRC), its specificity and sensitivity for AD were evaluated using datasets from other neurodegenerative disorders (DLB, FTD, PD, and non-AD). Furthermore, we investigated the association of the identified proteomic signature with the progression to dementia and the rate of memory decline. Finally, pathway and network enrichment analyses were performed separately for each protein group to gain mechanistic insights into AD pathophysiology (Fig. 1).

### ATN Classification

Amyloid-β (Aβ_42_) and hyperphosphorylated Tau 181 (pTau) biomarker levels obtained from CSF samples were utilized to categorize participants into cases and controls using the AT(N) classification framework^1^. This framework was applied separately for each individual cohort and independently for Aβ42 and pTau biomarkers, as described previously ^102,103^. Briefly, we utilized Gaussian mixture models to dichotomize quantitative Aβ42 and pTau measures into high (Biomarker positive) and low levels (Biomarker negative). Individuals with low CSF Aβ42 and high pTau levels were classified as amyloid/tau positive (A^+^T^+^), indicating high plaque and tangles in the brain. Conversely, individuals with high Aβ42 and low pTau levels were defined as controls (A^−^T^−^), indicating low plaque and tangles in the brain. Individuals with low CSF Aβ42 and pTau levels were classified as amyloid positive and tau negative (A^+^T^−^), indicating asymptomatic stages of AD characterized by high plaque and low tangles in the brain. Overall, the discovery cohorts that comprised Knight ADRC and FACE, contained 490 A^+^T^+^, 680 A^−^T^−^, and 284 A^+^T^−^ individuals, whereas, replication cohorts that consisted of ADNI and Barcelona-1, contained 358 A^+^T^+^, 235 A^−^T^−^, and 239 A^+^T^−^ individuals. Similarly, the completely independent replication dataset from Stanford ADRC cohorts contained 27 A^+^T^+^, 80 A^−^T^−^, and 25 A^+^T^−^ individuals (Fig. 2A).

### Proteomics data collection, processing and quality control (QC)

CSF samples in each cohort were collected through a lumbar puncture in the morning following an overnight fast. All samples underwent identical protocols for preparation and processing and were stored at −80 °C. To mitigate batch effects, the samples were sent together to SomaLogic and randomly allocated across different plates. Protein abundance levels were quantified using the SomaLogic aptamer-based SOMAscan platform that offers a multiplexed-based single-stranded DNA aptamer assay for protein quantification. The obtained data contains the quantitative levels of 7,293 aptamers measured in relative fluorescence unit (RFU). Initial data normalization was conducted by SomaLogic, which utilized hybridization controls for intra-plate and median signals to account for inter-plate variability ^104^. SomaLogic also performed an additional normalization step where data is further normalized against an external reference to control for biological variation ^105^. Aptamer– and individual–level QC were subsequently carried out for the detection and exclusion of outlier analytes and samples, using an in-house developed pipeline ^103,105^. Briefly, we removed all the aptamers with a maximum absolute difference between calibration and median scale factors surpassing 0.5, calculated individually for each plate. Additionally, we removed aptamers with a median coefficient of variation (CV) exceeding 0.15 or those that deviated beyond 1.5-fold of the interquartile range (IQR) on either end in over 85% of samples.

The IQR was calculated based on log10-transformed protein levels. At the end of aptamer-level QC, we also excluded analytes targeting non-human proteins. In the individual-level QC, a sample was removed if the log10-transformed RFU levels for that sample deviated beyond 1.5-fold of the IQR in over 85% of the aptamers. In total, 2,286 samples and 7,029 aptamers targeting 6,163 unique proteins passed the final QC and were used for subsequent analyses.

### Differential abundance analysis

Differential abundance of protein analytes across different AT groups (A^−^T^−^ vs. A^+^T^+^, A^−^T^−^ vs. A^+^T^−^, and A^+^T^−^ vs. A^+^T^+^) was detected using the following linear regression model where age at CSF draw, sex, plate id, and first two surrogate variables (SV) were used as covariates.


$${\text{log}}_{\text{10}}\left(\text{protein level}\right)\text{ \textasciitilde{} Status + age + sex + plate + SV1 + SV2}$$

We used “lm” function from the base stats package in R version 4.3.0 ^106^ for constructing the linear regression model and applied it to the log10 normalized protein analyte abundance data that follows a normal distribution. Status corresponds to the Binarized AT status (e.g., A^+^T^+^ = 1 and A^−^T^−^ = 0) of the individual. The computation of SV was carried out using “num.sv” function from the R sva package version 3.48.0 ^107^ with a random seed value fixed to 2022. P values corresponding to the significance of alteration of analytes in the comparison under investigation were corrected for false discovery rate (FDR) using “p.adjust” function from the base stats R package. The results of the differential abundance analysis in the form of significantly up- and down-regulated protein analytes were visualized in the form of a volcano plot using the EnhancedVolcano R function and package version 1.18.0 (RRID:SCR_018931).

Protein analytes that passed FDR correction (FDR < 0.05) in the stage 1 were further tested for differential protein expression in the stage 2 using the same linear regression model. Next, the analytes that also passed FDR correction (FDR < 0.05) in the stage 2 and showed a consistent direction of estimate (i.e., up- or down-regulated in both discovery and replication stages) were considered for meta-analysis. We employed Stouffer's Z method for performing the meta-analysis using the “combinePValues” function from *scran* R package version 1.28.1 ^108^. Stouffer's Z method ^109^ was used because of its inherent property of independence from test statistics that tends to prioritize symmetric rejection and is less affected by a single low p-value, thereby, requiring more consistently low p-values to yield a low combined p-value ^110^. A more stringent Bonferroni correction was applied to the meta-analysis p values using p.adjust function in R to identify a final set of significantly altered (Bonf < 0.05) protein analytes.

### Prediction models

Protein analytes that showed significant alterations in A^+^T^+^ individuals in comparison to A^−^T^−^ across both discovery (stage 1) and replication (stage 2) cohorts as well as in the meta-analysis (stage 3), were considered for building an AD prediction model. As the number of differentially abundant analytes was relatively high (n=2,173), we used least absolute shrinkage and selection operator (Lasso) regression model ^25^ with five-fold cross-validation to identify a minimum set of most informative proteins for developing the AD prediction model. We used “train” function in the caret R package version 6.0–94 ^111^ for employing the Lasso regression model in the stage 1 training dataset (n=819). In the case of highly correlated (Pearson correlation > 0.8) analytes, one of the representative analytes was kept from each pair. Starting from an initial set of 2,173 differentially abundant analytes, we identified a subset of 38 analytes that comprised our initial CSF AD proteomic signature. Because we also aimed to test the performance of this prediction model in an external dataset profiled using a different platform (Stanford ADRC), we retained an overlapping set of proteins (n=25) within both datasets for subsequent analysis. After examining the association of this proteomic panel with AT status in the stage 1 training data, we identified a group of 11 proteins that displayed significant associations (P < 0.05), constituting our distinctive AD-specific CSF proteomic signature.

To assess the predictive power of the proposed AD proteomic signature, we used a three-stage (discovery, replication, and validation) approach. The identified 11-protein AD prediction model was trained using 70% of stage 1 training data (discovery) and tested on the remaining 30% of the stage 1 testing data as well as the complete stage 2 data (replication) using the model weights (cutoffs) derived from stage 1 training. Finally, we tested the model performance in a completely independent validation dataset from the Stanford ADRC cohort, which, unlike our stage 1 and stage 2 cohorts, used the 5K Somascan panel for proteome profiling. Although this prediction model was inferred using the AT framework, its performance was also tested on the data where individuals were stratified using clinical case-control diagnosis based on the clinical dementia rating (CDR^©^) and cognitive assessment. Furthermore, the specificity of this AD-specific prediction model was also assessed in datasets from other dementias, such as dementia Lewy body (DLB), frontotemporal dementia (FTD), and Parkinson’s disease (PD) as well as other non-AD individuals. For PD, we used the CSF proteomics dataset obtained from the Parkinson's Progression Markers Initiative (PPMI) study ^112^ that included 507 PD and 168 control individuals, profiled using Somascan 5K panel. The sensitivity (true-positive rate) and specificity (true-negative rate) of the developed AD prediction model were assessed by plotting the receiver operator characteristic (ROC) curves using pROC R package version 1.18.2 ^113^. To further evaluate the performance of these proteins, we generated areas under the curves (AUC) statistics and also estimated the positive predictive value (PPV) and negative predictive value (NPV) based on Youden’s J statistic ^114^ optimal cut-off using “cords” function in the pROC R package.

### AD CSF proteome clustering

A total of 2,173 protein analytes that showed significant alterations in the A^+^T^+^ compared to A^−^T^−^ individuals were clustered into 4 distinct groups based on their estimates (direction of effects) and significance (p-value) across three different stages in the AD continuum (A^−^T^−^, A^+^T^−^, A^+^T^+^). A pair-wise differential abundance analysis (DAA) was performed between all these AT groups (A^−^T^−^ vs. A^+^T^−^, A^+^T^−^ vs. A^+^T^+^, and A^−^T^−^ vs. A^+^T^+^) to track the trajectory of protein abundance from control (A^−^T^−^) to asymptomatic (A^+^T^−^) stage leading to AD (A^+^T^+^). For instance, if a protein showed significant alterations (p-value < 0.05) between A^−^T^−^ and A^+^T^−^ as well as between A^+^T^−^ and A^+^T^+^ with a positive estimate (β > 0) in both comparisons, we considered this protein to be increasing linearly across the AD continuum. Following this rationale, we detected 4 major trajectories: i) proteins going up linearly (group 1), ii) proteins going up from A^−^T^−^ to A^+^T^−^ but then going down from A^+^T^−^ to A^+^T^+^ (group 2), iii) proteins going linearly down (group 3), and proteins that go down from A^−^T^−^ to A^+^T^−^ but then go up from A^+^T^−^ to A^+^T^+^ (group 4). These groups contained different numbers of protein analytes ranging from 190 (group 3) to 1086 (group 4) that were further analyzed using pathway enrichment and network analyses (Fig. 4A).

### Pathway enrichment analysis

We performed functional enrichment analysis separately in each of the identified protein groups using ClusterProfiler R package version 4.8.1 ^115^. Since different groups contained widely variable numbers of protein analytes e.g., group 4 (G4) having 1086 and group 3 (G3) having 190 analytes, we used a network-based approach for the pre-selection of topologically important proteins. Briefly, a differential network-based approach ^116^ was employed for building networks of proteins in each of the groups based on the manually curated regulatory interaction retrieved from Metacore (Clarivate Analytics) database (RRID:SCR_008125). This prior-knowledge network was further pruned to remove interactions that were not compatible with Booleanized protein abundance data where proteins with positive and negative estimates in the A^+^T^+^ vs. A^−^T^−^ DAA comparison were considered as 1 and 0, respectively. As a result, we obtained two condition-specific networks compatible with protein abundance signatures in the A^+^T^+^ and A^−^T^−^ individuals. Next, we used “clusters” function from the igraph R package (version 1.4.23) ^117^ to detect all elementary network circuits in the network representing A^+^T^+^ phenotype. An elementary circuit is a path that begins and ends at the same node while visiting each intermediate node only once along the way. These elementary circuits comprise positive (i.e., circuit with an even number of inhibitions) and negative (i.e., circuit with an odd number of inhibitions) circuits that have been shown to play a crucial role in maintaining network stability ^118–120^. Finally, the proteins that constitute these elementary circuits were used for performing the functional enrichment analysis, separately in each of the four identified groups. We used “enrichKEGG” function to perform the Kyoto Encyclopedia of Genes and Genomes (KEGG) enrichment analysis of a gene set of interest where all of the proteins in the Somascan panel (n=6,163) were used as the background. The only exception was group 3, where gene ontology (GO) enrichment analysis was conducted and unadjusted p-value were reported due to a limited number of proteins in that group. The significance of functional enrichment analysis was reported as the p-value of the hypergeometric test for over-representation, followed by adjustment for FDR in testing multiple hypotheses. We considered results with FDR < 0.05 as statistically significant and the top 10 pathways were shown in the form of a barplot using “ggplot” function from the ggplot2 R package version 3.4.2 ^121^.

### Protein network analysis

Functional interaction networks were built to understand the cross-talk between key proteins present in each of the four identified groups. Specifically, proteins belonging to the top 10 pathways from the KEGG and GO enrichment analyses were used to build protein-protein interaction (PPI) network using STRING database version 12.0 ^55^. To obtain an appropriate set of functional PPI between the identified set of proteins, our analysis was restricted to Homo sapiens with active interaction sources from “Experiments”, “Databases”, and “Co-expression”. The only exception was group 3, where all of the available sources including “Text-mining”, “Neighborhood”, “Gene Fusion”, and “Co-occurrence” were also considered because of the limited number of proteins in that group. The resulting functional PPIs were visually shown in the form of a network developed by the Cytoscape tool version 3.10.0 ^122^.

### Cell type enrichment analysis

For conducting cell type enrichment analysis, we used an in-house developed and manually curated marker list that was prepared using the CellMarker database and existing literature ^123,124^. As the CellMarker database does not provide cell-type-specific marker information, since many marker genes are associated with multiple cell types, we used existing literature to manually curate a list of marker genes that are exclusively expressed in only one particular cell type. A hypergeometric test ^125^, which is equivalent to one-tailed Fisher’s exact test, was employed for performing the cell type enrichment analysis using “phyper” function in base R package stats.

### Progression to dementia and time-to-event analysis

In order to assess if there is a significant difference in AD progression between predicted proteomic signature–positive and –negative individuals, the rate of dementia progression analysis was performed using CDR sum of boxes (CDR-SB) per year, as described previously ^90^. As longitudinal data to assess change in CDR-SB was available for only Knight ADRC and ADNI cohorts, we focused on investigating the differences between the rate of dementia progression for individuals predicted to be proteomic signature–positive and –negative according to 11 analytes AD prediction model. This analysis was performed on longitudinal data from 333 individuals in the Knight ADRC (n=117; A^−^T^−^ = 23, A^+^T^+^ = 94) and ADNI (n=215; A^−^T^−^ = 81, A^+^T^+^ = 135) cohorts. A linear regression model was fit, regressing CDR-SB on time in years, where age, sex, predicted biomarker status, known AT status, and initial CDR were used as covariates, as previously explained ^90^. The model also included interaction terms between time and predicted status as well as age and predicted status.

We also conducted a time-to-event analysis for individuals predicted to be proteomic signature–positive and –negative using Cox proportional hazards regression model ^92^ implemented in “survfit” function of the *survival* R package (version 3.5.5, RRID:SCR_021137). At first, we created a survival object using the “Surv” function that used follow-up years and censored status and resulted in a response variable that was further regressed on predicted biomarker status to compute an estimate of a survival curve for censored data using the Kaplan-Meier method ^126^. We used “ggsurvplot” function from the survminer R package (version 0.4.9, RRID:SCR_021094) to visualize the Kaplan-Meier plots for probability of not developing AD over a 15-year time period.

## Supplementary Material

Supplement 1

## Figures and Tables

**Table 1:** Demographics information of participants at the time of the CSF draw.

	Stage 1 (Discovery)		Stage 2 (Replication)		Validation
Cohort	Knight ADRC	FACE	ADNI	Barcelona-1	Stanford ADRC
**Sample size**	836	618	700	132	132
**Females (%)**	54.31	59.22	42.86	54.55	55.30
**Males (%)**	45.69	40.78	57.14	45.45	44.70
**Age (mean)**	70.80	72.14	73.49	68.16	68.70
**Age (SD)**	8.51	8.44	7.50	8.19	7.55
**A**^**+**^**T**^**+**^ **(%)**	20.45	51.62	40.00	59.09	20.45
**A**^**+**^**T**^**−**^ **(%)**	55.62	34.79	31.43	11.36	18.94
**A**^**−**^**T**^**−**^ **(%)**	23.92	13.59	28.57	29.55	60.61
**APOE4+ (%)**	39.00	26.70	50.43	50.00	46.21

This table summarizes basic demographic information of CSF proteomics study participants. For each cohort, we report sample size, percentage of females and males, mean age and its standard deviation (SD), percentage of A+T+, A+T**−**, and A**−**T**−** participants, and percentage of APOE4+ and APOE4**−** individuals. Abbreviations: Knight-ADRC, Knight Alzheimer’s Disease Research Center; ADNI, Alzheimer's Disease Neuroimaging Initiative; SD, standard deviation.

## List of abbreviations

A: Amyloid pathology
AD: Alzheimer’s disease
ADNI: Alzheimer's Disease Neuroimaging Initiative
APOE: Apolipoprotein E
AUC: Area under the curve
Aβ: Amyloid beta
BDNF: Brain-Derived Neurotrophic Factor
CDR©: Clinical dementia rating
CDR-SB: Clinical dementia rating sum of boxes
CES1: Carboxylesterase 1
CNS: Central nervous system
CO: Controls
CSF: Cerebrospinal fluid
CTSD: Cathepsin D
DAA: Differentially abundant analytes
DLB: Dementia lewy body
FACE: Fundació ACE Alzheimer Center
FDR: False discovery rate
FOXO1: Forkhead Box O1
FOXO3: Forkhead Box O3
FTD: Frontotemporal dementia
GFAP: Glial Fibrillary Acidic Protein
GO: Gene ontology
GRN: Granulin Precursor
IL6: Interleukin 6
KEGG: Kyoto Encyclopedia of Genes and Genomes
Knight-ADRC: Knight Alzheimer’s Disease Research Center
LOAD: Late-onset AD
MCI: Mild cognitive impairment
N: Neurodegeneration
NCK2: NCK Adaptor Protein 2
NEFL: Neurofilament Light Chain
NFKB1: Nuclear Factor Kappa B Subunit 1
NFL: Neurofilament light chain
NIA-AA: National Institute on Aging and Alzheimer's Association
NPV: Negative predictive value
PD: Parkinson’s disease
PPI: Protein-protein interaction
PPP3R1: Protein Phosphatase 3 Regulatory Subunit B, Alpha
PPV: Positive predictive value
pTau: Hyperphosphorylated Tau 181
PTK2B: Protein Tyrosine Kinase 2 Beta
QC: Quality control
SHARPIN: SHANK Associated RH Domain Interactor
SMOC1: SPARC-related modular calcium-binding protein 1
SNCA: Alpha-Synuclein
Stanford ADRC: Stanford Alzheimer Disease Research Center
STAT1: Signal transducer and activator of transcription-1
SVs: Surrogate variables
T: Tau pathology
TMT: Tandem mass tag
TREM2: Triggering Receptor Expressed On Myeloid Cells 2

## References

[1] Jack C. R. J. A/T/N: An unbiased descriptive classification scheme for Alzheimer disease biomarkers. Neurology 87, 539–547 (2016). 27371494 10.1212/WNL.0000000000002923 PMC4970664

[2] Gatz M. Role of genes and environments for explaining Alzheimer disease. Archives of general psychiatry 63, 168–174 (2006). 16461860 10.1001/archpsyc.63.2.168

[3] Karch C. M. & Goate A. M. Alzheimer’s disease risk genes and mechanisms of disease pathogenesis. Biological psychiatry 77, 43–51 (2015). 24951455 10.1016/j.biopsych.2014.05.006 PMC4234692

[4] Bagyinszky E., Giau V. Van & An S. A. Transcriptomics in Alzheimer’s Disease: Aspects and Challenges. International journal of molecular sciences 21, (2020). 10.3390/ijms21103517 PMC7278930 32429229

[5] Yang H.-S. Genetics of Gene Expression in the Aging Human Brain Reveal TDP-43 Proteinopathy Pathophysiology. Neuron 107, 496–508.e6 (2020). 32526197 10.1016/j.neuron.2020.05.010 PMC7416464

[6] Park S. A., Han S. M. & Kim C. E. New fluid biomarkers tracking non-amyloid-β and non-tau pathology in Alzheimer’s disease. Experimental & molecular medicine 52, 556–568 (2020). 32284537 10.1038/s12276-020-0418-9 PMC7210893

[7] Olsson B. CSF and blood biomarkers for the diagnosis of Alzheimer’s disease: a systematic review and meta-analysis. The Lancet. Neurology 15, 673–684 (2016). 27068280 10.1016/S1474-4422(16)00070-3

[8] Kouzarides T. Chromatin Modifications and Their Function. Cell Preprint at 10.1016/j.cell.2007.02.005 (2007). 17320507

[9] Buchhave P. Cerebrospinal fluid levels of β-amyloid 1–42, but not of tau, are fully changed already 5 to 10 years before the onset of Alzheimer dementia. Archives of general psychiatry 69, 98–106 (2012). 22213792 10.1001/archgenpsychiatry.2011.155

[10] Jack C. R. J. NIA-AA Research Framework: Toward a biological definition of Alzheimer’s disease. Alzheimer’s & dementia : the journal of the Alzheimer’s Association 14, 535–562 (2018). 10.1016/j.jalz.2018.02.018 PMC5958625 29653606

[11] Schoonenboom N. S. M. Cerebrospinal fluid markers for differential dementia diagnosis in a large memory clinic cohort. Neurology 78, 47–54 (2012). 22170879 10.1212/WNL.0b013e31823ed0f0

[12] Ewers M. CSF biomarkers for the differential diagnosis of Alzheimer’s disease: A large-scale international multicenter study. Alzheimer’s & dementia : the journal of the Alzheimer’s Association 11, 1306–1315 (2015). 10.1016/j.jalz.2014.12.006 25804998

[13] Robinson J. L. Neurodegenerative disease concomitant proteinopathies are prevalent, age-related and APOE4-associated. Brain : a journal of neurology 141, 2181–2193 (2018). 29878075 10.1093/brain/awy146 PMC6022546

[14] Shi L. Multiomics profiling of human plasma and cerebrospinal fluid reveals ATN-derived networks and highlights causal links in Alzheimer’s disease. Alzheimer’s & dementia : the journal of the Alzheimer’s Association 19, 3350–3364 (2023). 10.1002/alz.12961 36790009

[15] Johnson E. C. B. Large-scale deep multi-layer analysis of Alzheimer’s disease brain reveals strong proteomic disease-related changes not observed at the RNA level. Nature neuroscience 25, 213–225 (2022). 35115731 10.1038/s41593-021-00999-y PMC8825285

[16] Del Campo M. CSF proteome profiling across the Alzheimer’s disease spectrum reflects the multifactorial nature of the disease and identifies specific biomarker panels. Nature aging 2, 1040–1053 (2022). 37118088 10.1038/s43587-022-00300-1 PMC10292920

[17] Johnson E. C. B. Large-scale proteomic analysis of Alzheimer’s disease brain and cerebrospinal fluid reveals early changes in energy metabolism associated with microglia and astrocyte activation. Nature medicine 26, 769–780 (2020). 10.1038/s41591-020-0815-6 PMC7405761 32284590

[18] Higginbotham L. Integrated proteomics reveals brain-based cerebrospinal fluid biomarkers in asymptomatic and symptomatic Alzheimer’s disease. Science advances 6, (2020). 10.1126/sciadv.aaz9360 PMC7577712 33087358

[19] Sung Y. J. Proteomics of brain, CSF, and plasma identifies molecular signatures for distinguishing sporadic and genetic Alzheimer’s disease. Science translational medicine 15, eabq5923 (2023). 37406134 10.1126/scitranslmed.abq5923 PMC10803068

[20] Benson M. D., Ngo D., Ganz P. & Gerszten R. E. Emerging Affinity Reagents for High Throughput Proteomics: Trust, but Verify. Circulation 140, 1610–1612 (2019). 31710523 10.1161/CIRCULATIONAHA.119.039912 PMC7577374

[21] Fagan A. M. Cerebrospinal fluid tau/beta-amyloid(42) ratio as a prediction of cognitive decline in nondemented older adults. Archives of neurology 64, 343–349 (2007). 17210801 10.1001/archneur.64.3.noc60123

[22] Modeste E. S. Quantitative proteomics of cerebrospinal fluid from African Americans and Caucasians reveals shared and divergent changes in Alzheimer’s disease. Molecular neurodegeneration 18, 48 (2023). 37468915 10.1186/s13024-023-00638-z PMC10355042

[23] Bader J. M. Proteome profiling in cerebrospinal fluid reveals novel biomarkers of Alzheimer’s disease. Molecular systems biology 16, e9356 (2020). 32485097 10.15252/msb.20199356 PMC7266499

[24] Tijms B. M. Pathophysiological subtypes of Alzheimer’s disease based on cerebrospinal fluid proteomics. Brain : a journal of neurology 143, 3776–3792 (2020). 33439986 10.1093/brain/awaa325 PMC7805814

[25] Tibshirani R. Regression Shrinkage and Selection Via the Lasso. Journal of the Royal Statistical Society: Series B (Methodological) 58, 267–288 (1996).

[26] Wang L., Zhou Y., Chen D. & Lee T. H. Peptidyl-Prolyl Cis/Trans Isomerase Pin1 and Alzheimer’s Disease. Frontiers in cell and developmental biology 8, 355 (2020). 32500074 10.3389/fcell.2020.00355 PMC7243138

[27] Vega I. E., Umstead A., Wygant C. M., Beck J. S. & Counts S. E. Ezrin Expression is Increased During Disease Progression in a Tauopathy Mouse Model and Alzheimer’s Disease. Current Alzheimer research 15, 1086–1095 (2018). 30101710 10.2174/1567205015666180813152043 PMC6522142

[28] Roberts J. A. Unbiased proteomics and multivariable regularized regression techniques identify SMOC1, NOG, APCS, and NTN1 in an Alzheimer’s disease brain proteomic signature. npj Aging 9, 18 (2023). 37414805 10.1038/s41514-023-00112-6 PMC10326005

[29] Giacomucci G. Plasma neurofilament light chain as a biomarker of Alzheimer’s disease in Subjective Cognitive Decline and Mild Cognitive Impairment. Journal of neurology 269, 4270–4280 (2022). 35288777 10.1007/s00415-022-11055-5 PMC9293849

[30] Kim K. Y., Shin K. Y. & Chang K.-A. GFAP as a Potential Biomarker for Alzheimer’s Disease: A Systematic Review and Meta-Analysis. Cells 12, (2023). 10.3390/cells12091309 PMC10177296 37174709

[31] Sleegers K., Brouwers N. & Van Broeckhoven C. Role of progranulin as a biomarker for Alzheimer’s disease. Biomarkers in medicine 4, 37–50 (2010). 20387302 10.2217/bmm.09.82

[32] Zhou Z. Integrative genomic analysis of PPP3R1 in Alzheimer’s disease: a potential biomarker for predictive, preventive, and personalized medical approach. The EPMA journal 12, 647–658 (2021). 34956428 10.1007/s13167-021-00261-2 PMC8648944

[33] Shim K. H., Kang M. J., Youn Y. C., An S. S. A. & Kim S. Alpha-synuclein: a pathological factor with Aβ and tau and biomarker in Alzheimer’s disease. Alzheimer’s Research & Therapy 14, 201 (2022). 10.1186/s13195-022-01150-0 PMC9805257 36587215

[34] Bellenguez C. New insights into the genetic etiology of Alzheimer’s disease and related dementias. Nature Genetics 54, 412–436 (2022). 35379992 10.1038/s41588-022-01024-z PMC9005347

[35] Bai B. Deep Multilayer Brain Proteomics Identifies Molecular Networks in Alzheimer’s Disease Progression. Neuron 105, 975–991.e7 (2020). 31926610 10.1016/j.neuron.2019.12.015 PMC7318843

[36] Johnson E. C. B. Cerebrospinal fluid proteomics define the natural history of autosomal dominant Alzheimer’s disease. Nature Medicine 29, 1979–1988 (2023). 10.1038/s41591-023-02476-4 PMC10427428 37550416

[37] Mori Y. Serum BDNF as a Potential Biomarker of Alzheimer’s Disease: Verification Through Assessment of Serum, Cerebrospinal Fluid, and Medial Temporal Lobe Atrophy. Frontiers in neurology 12, 653267 (2021). 33967943 10.3389/fneur.2021.653267 PMC8102980

[38] Gao L., Zhang Y., Sterling K. & Song W. Brain-derived neurotrophic factor in Alzheimer’s disease and its pharmaceutical potential. Translational Neurodegeneration 11, 4 (2022). 35090576 10.1186/s40035-022-00279-0 PMC8796548

[39] Kim J.-W. Identification of Cathepsin D as a Plasma Biomarker for Alzheimer’s Disease. Cells 10, (2021). 10.3390/cells10010138 PMC7827175 33445607

[40] Chai Y. L. Lysosomal cathepsin D is upregulated in Alzheimer’s disease neocortex and may be a marker for neurofibrillary degeneration. Brain pathology (Zurich, Switzerland) 29, 63–74 (2019). 30051532 10.1111/bpa.12631 PMC8028263

[41] Jones S. V. & Kounatidis I. Nuclear Factor-Kappa B and Alzheimer Disease, Unifying Genetic and Environmental Risk Factors from Cell to Humans. Frontiers in immunology 8, 1805 (2017). 29312321 10.3389/fimmu.2017.01805 PMC5732234

[42] Jha N. K. Nuclear factor-kappa β as a therapeutic target for Alzheimer’s disease. Journal of Neurochemistry 150, 113–137 (2019). 30802950 10.1111/jnc.14687

[43] Cacabelos R. Personalized Management and Treatment of Alzheimer’s Disease. Life (Basel, Switzerland) 12, (2022). 10.3390/life12030460 PMC8951963 35330211

[44] Lyra e Silva N. M. Pro-inflammatory interleukin-6 signaling links cognitive impairments and peripheral metabolic alterations in Alzheimer’s disease. Translational Psychiatry 11, 251 (2021). 33911072 10.1038/s41398-021-01349-z PMC8080782

[45] Cojocaru I. M., Cojocaru M., Miu G. & Sapira V. Study of interleukin-6 production in Alzheimer’s disease. Romanian journal of internal medicine = Revue roumaine de medecine interne 49, 55–58 (2011). 22026253

[46] Liu L. Cross-Talking Pathways of Forkhead Box O1 (FOXO1) Are Involved in the Pathogenesis of Alzheimer’s Disease and Huntington’s Disease. Oxidative medicine and cellular longevity 2022, 7619255 (2022). 35154571 10.1155/2022/7619255 PMC8831070

[47] Du S. & Zheng H. Role of FoxO transcription factors in aging and age-related metabolic and neurodegenerative diseases. Cell & Bioscience 11, 188 (2021). 34727995 10.1186/s13578-021-00700-7 PMC8561869

[48] Morenas-Rodríguez E. Soluble TREM2 in CSF and its association with other biomarkers and cognition in autosomal-dominant Alzheimer’s disease: a longitudinal observational study. The Lancet. Neurology 21, 329–341 (2022). 35305339 10.1016/S1474-4422(22)00027-8 PMC8926925

[49] Liu C.-C., Liu C.-C., Kanekiyo T., Xu H. & Bu G. Apolipoprotein E and Alzheimer disease: risk, mechanisms and therapy. Nature reviews. Neurology vol. 9 106–118 Preprint at 10.1038/nrneurol.2012.26 (2013). 23296339 PMC3726719

[50] Liu W. Neurogranin as a cognitive biomarker in cerebrospinal fluid and blood exosomes for Alzheimer’s disease and mild cognitive impairment. Translational Psychiatry 10, 125 (2020). 32350238 10.1038/s41398-020-0801-2 PMC7190828

[51] Casaletto K. B. Neurogranin, a synaptic protein, is associated with memory independent of Alzheimer biomarkers. Neurology 89, 1782–1788 (2017). 28939668 10.1212/WNL.0000000000004569 PMC5664306

[52] Qian M., Shen X. & Wang H. The Distinct Role of ADAM17 in APP Proteolysis and Microglial Activation Related to Alzheimer’s Disease. Cellular and molecular neurobiology 36, 471–482 (2016). 26119306 10.1007/s10571-015-0232-4 PMC11482503

[53] Hartl D. A rare loss-of-function variant of ADAM17 is associated with late-onset familial Alzheimer disease. Molecular Psychiatry 25, 629–639 (2020). 29988083 10.1038/s41380-018-0091-8 PMC7042727

[54] Cruchaga C. Proteogenomic analysis of human cerebrospinal fluid identifies neurologically relevant regulation and informs causal proteins for Alzheimer’s disease. Research square Preprint at 10.21203/rs.3.rs-2814616/v1 (2023). PMC11831731 39528825

[55] Szklarczyk D. The STRING database in 2021: customizable protein-protein networks, and functional characterization of user-uploaded gene/measurement sets. Nucleic acids research 49, D605–D612 (2021). 33237311 10.1093/nar/gkaa1074 PMC7779004

[56] Cruchaga C. SNPs Associated with Cerebrospinal Fluid Phospho-Tau Levels Influence Rate of Decline in Alzheimer’s Disease. PLoS Genet 6, e1001101- (2010). 20862329 10.1371/journal.pgen.1001101 PMC2940763

[57] Mirzaa G., Foss K., Nattakom M. & Chung W. K. PPP2R5D-Related Neurodevelopmental Disorder. GeneReviews^®^ [Internet]. Seattle (WA): University of Washington, Seattle; 1993–2020. (2019). 30676711

[58] Dube U. An atlas of cortical circular RNA expression in Alzheimer disease brains demonstrates clinical and pathological associations. Nat Neurosci (2019) doi:10.1038/s41593-019-0501-5. PMC6858549 31591557

[59] Stefanis L. α-Synuclein in Parkinson’s disease. Cold Spring Harbor perspectives in medicine 2, a009399 (2012). 22355802 10.1101/cshperspect.a009399 PMC3281589

[60] Heremans I. P. Parkinson’s disease protein PARK7 prevents metabolite and protein damage caused by a glycolytic metabolite. Proceedings of the National Academy of Sciences of the United States of America 119, (2022). 10.1073/pnas.2111338119 PMC8795555 35046029

[61] Didonna A. & Opal P. The role of neurofilament aggregation in neurodegeneration: lessons from rare inherited neurological disorders. Molecular neurodegeneration 14, 19 (2019). 31097008 10.1186/s13024-019-0318-4 PMC6524292

[62] Bomont P. The dazzling rise of neurofilaments: Physiological functions and roles as biomarkers. Current opinion in cell biology 68, 181–191 (2021). 33454158 10.1016/j.ceb.2020.10.011

[63] Sung Y. J. Proteomics of brain, CSF, and plasma identifies molecular signatures for distinguishing sporadic and genetic Alzheimer’s disease. Sci Transl Med 15, eabq5923 (2023). 37406134 10.1126/scitranslmed.abq5923 PMC10803068

[64] Potter H. Review and hypothesis: Alzheimer disease and Down syndrome--chromosome 21 nondisjunction may underlie both disorders. American journal of human genetics 48, 1192–1200 (1991). 1827946 PMC1683102

[65] Pimenova A. A. Alzheimer’s-associated PU.1 expression levels regulate microglial inflammatory response. Neurobiology of disease 148, 105217 (2021). 33301878 10.1016/j.nbd.2020.105217 PMC7808757

[66] Huang K.-L. A common haplotype lowers PU.1 expression in myeloid cells and delays onset of Alzheimer’s disease. Nature neuroscience 20, 1052–1061 (2017). 28628103 10.1038/nn.4587 PMC5759334

[67] Rustenhoven J. PU.1 regulates Alzheimer’s disease-associated genes in primary human microglia. Molecular neurodegeneration 13, 44 (2018). 30124174 10.1186/s13024-018-0277-1 PMC6102813

[68] Daver N., Schlenk R. F., Russell N. H. & Levis M. J. Targeting FLT3 mutations in AML: review of current knowledge and evidence. Leukemia 2019 33:2 33, 299–312 (2019). 30651634 10.1038/s41375-018-0357-9 PMC6365380

[69] Pandolfi P. P. Oncogenes and tumor suppressors in the molecular pathogenesis of acute promyelocytic leukemia. Hum Mol Genet 10, 769–775 (2001). 11257111 10.1093/hmg/10.7.769

[70] Nanri T. A family harboring a germ-line N-terminal C/EBPα mutation and development of acute myeloid leukemia with an additional somatic C-terminal C/EBPα mutation. Genes Chromosomes Cancer 49, 237–241 (2010). 19953636 10.1002/gcc.20734

[71] Kitamura Y. Alteration of transcription factors NF-kappaB and STAT1 in Alzheimer’s disease brains. Neuroscience letters 237, 17–20 (1997). 9406869 10.1016/s0304-3940(97)00797-0

[72] Ju Hwang C., Choi D.-Y., Park M. H. & Hong J. T. NF-κB as a Key Mediator of Brain Inflammation in Alzheimer’s Disease. CNS & neurological disorders drug targets 18, 3–10 (2019). 28782486 10.2174/1871527316666170807130011

[73] Pradhan R. Serum FOXO3A: A ray of hope for early diagnosis of Alzheimer’s disease. Mechanisms of ageing and development 190, 111290 (2020). 32603667 10.1016/j.mad.2020.111290

[74] Li T., Chen X., Zhang C., Zhang Y. & Yao W. An update on reactive astrocytes in chronic pain. Journal of neuroinflammation 16, 140 (2019). 31288837 10.1186/s12974-019-1524-2 PMC6615111

[75] Singh S. Axin-2 knockdown promote mitochondrial biogenesis and dopaminergic neurogenesis by regulating Wnt/β-catenin signaling in rat model of Parkinson’s disease. Free Radical Biology and Medicine 129, 73–87 (2018). 30176346 10.1016/j.freeradbiomed.2018.08.033

[76] Jennings R., Kelliher M. & O’Neill C. Markedly reduced levels of soluble β-catenin in Alzheimer’s disease brain. Biochemical Society Transactions 28, A35–A35 (2000).

[77] Ghanevati M. & Miller C. A. Phospho-beta-catenin accumulation in Alzheimer’s disease and in aggresomes attributable to proteasome dysfunction. J Mol Neurosci 25, 79–94 (2005). 15781969 10.1385/JMN:25:1:079

[78] Roichman A. Restoration of energy homeostasis by SIRT6 extends healthy lifespan. Nature Communications 2021 12:1 12, 1–18 (2021). 10.1038/s41467-021-23545-7 PMC8163764 34050173

[79] Kanfi Y. The sirtuin SIRT6 regulates lifespan in male mice. Nature 2012 483:7388 483, 218–221 (2012). 22367546 10.1038/nature10815

[80] Pons V., Lévesque P., Plante M.-M. & Rivest S. Conditional genetic deletion of CSF1 receptor in microglia ameliorates the physiopathology of Alzheimer’s disease. Alzheimers Res Ther 13, 8 (2021). 33402196 10.1186/s13195-020-00747-7 PMC7783991

[81] Folch J. The Implication of the Brain Insulin Receptor in Late Onset Alzheimer’s Disease Dementia. Pharmaceuticals (Basel) 11, (2018). 10.3390/ph11010011 PMC5874707 29382127

[82] Green K. N., Crapser J. D. & Hohsfield L. A. To Kill a Microglia: A Case for CSF1R Inhibitors. Trends Immunol 41, 771–784 (2020). 32792173 10.1016/j.it.2020.07.001 PMC7484341

[83] Snow A. D. & Wight T. N. Proteoglycans in the pathogenesis of Alzheimer’s disease and other amyloidoses. Neurobiology of aging 10, 481–497 (1989). 2682326 10.1016/0197-4580(89)90108-5

[84] Del-Aguila J. L. TREM2 brain transcript-specific studies in AD and TREM2 mutation carriers. Molecular neurodegeneration 14, 18 (2019). 31068200 10.1186/s13024-019-0319-3 PMC6505298

[85] Cruchaga C. Rare coding variants in the phospholipase D3 gene confer risk for Alzheimer’s disease. Nature 505, 550–554 (2014). 24336208 10.1038/nature12825 PMC4050701

[86] Lopera F. Resilience to autosomal dominant Alzheimer’s disease in a Reelin-COLBOS heterozygous man. Nat Med 29, 1243–1252 (2023). 37188781 10.1038/s41591-023-02318-3 PMC10202812

[87] Jain A. P. & Sathe G. Proteomics Landscape of Alzheimer’s Disease. Proteomes 9, (2021). 10.3390/proteomes9010013 PMC8005944 33801961

[88] Agrawal M. & Biswas A. Molecular diagnostics of neurodegenerative disorders. Frontiers in molecular biosciences 2, 54 (2015). 26442283 10.3389/fmolb.2015.00054 PMC4585189

[89] Serdar C. C., Cihan M., Yücel D. & Serdar M. A. Sample size, power and effect size revisited: simplified and practical approaches in pre-clinical, clinical and laboratory studies. Biochemia medica 31, 10502 (2021). 10.11613/BM.2021.010502 PMC7745163 33380887

[90] Del-Aguila J. L. Assessment of the Genetic Architecture of Alzheimer’s Disease Risk in Rate of Memory Decline. Journal of Alzheimer’s disease: JAD 62, 745–756 (2018). 29480181 10.3233/JAD-170834 PMC5989565

[91] Lin K. A. Marked gender differences in progression of mild cognitive impairment over 8 years. Alzheimer’s & dementia (New York, N. Y.) 1, 103–110 (2015). 10.1016/j.trci.2015.07.001 PMC4593067 26451386

[92] Harrell F. E. Cox Proportional Hazards Regression Model BT - Regression Modeling Strategies: With Applications to Linear Models, Logistic Regression, and Survival Analysis. in (ed. Harrell F. E.) 465–507 (Springer New York, 2001). doi:10.1007/978-1-4757-3462-1_19.

[93] Dhiman K. Cerebrospinal fluid neurofilament light concentration predicts brain atrophy and cognition in Alzheimer’s disease. Alzheimer’s & Dementia: Diagnosis, Assessment & Disease Monitoring 12, e12005 (2020). 10.1002/dad2.12005 PMC7085283 32211500

[94] Steinacker P. Neurofilaments in the diagnosis of motoneuron diseases: a prospective study on 455 patients. Journal of neurology, neurosurgery, and psychiatry 87, 12–20 (2016). 26296871 10.1136/jnnp-2015-311387

[95] Brettschneider J., Petzold A., Süssmuth S. D., Ludolph A. C. & Tumani H. Axonal damage markers in cerebrospinal fluid are increased in ALS. Neurology 66, 852–856 (2006). 16567701 10.1212/01.wnl.0000203120.85850.54

[96] Brettschneider J. The neurofilament heavy chain (NfH) in the cerebrospinal fluid diagnosis of Alzheimer’s disease. Dementia and geriatric cognitive disorders 21, 291–295 (2006). 16484807 10.1159/000091436

[97] Lutz M. W. & Chiba-Falek O. Bioinformatics pipeline to guide late-onset Alzheimer’s disease (LOAD) post-GWAS studies: Prioritizing transcription regulatory variants within LOAD-associated regions. Alzheimer’s & dementia (New York, N. Y.) 8, e12244 (2022). 10.1002/trc2.12244 PMC8864953 35229021

[98] Dumbacher M. Modifying Rap1-signalling by targeting Pde6δ is neuroprotective in models of Alzheimer’s disease. Molecular Neurodegeneration 13, 50 (2018). 30257685 10.1186/s13024-018-0283-3 PMC6158915

[99] LaFerla F. M. Calcium dyshomeostasis and intracellular signalling in alzheimer’s disease. Nature Reviews Neuroscience 3, 862–872 (2002). 12415294 10.1038/nrn960

[100] Elman J. A. Neural compensation in older people with brain amyloid-β deposition. Nature Neuroscience 17, 1316–1318 (2014). 25217827 10.1038/nn.3806 PMC4177011

[101] Freudenberg-Hua Y., Li W. & Davies P. The Role of Genetics in Advancing Precision Medicine for Alzheimer’s Disease-A Narrative Review. Frontiers in medicine 5, 108 (2018). 29740579 10.3389/fmed.2018.00108 PMC5928202

[102] Ali M. Leveraging large multi-center cohorts of Alzheimer Disease endophenotypes to understand the role of Klotho heterozygosity on disease risk. Preprint at 10.1101/2021.10.07.21264646 (2021). PMC9135221 35617280

[103] Timsina J. Comparative Analysis of Alzheimer’s Disease Cerebrospinal Fluid Biomarkers Measurement by Multiplex SOMAscan Platform and Immunoassay-Based Approach. Journal of Alzheimer’s disease: JAD 89, 193–207 (2022). 35871346 10.3233/JAD-220399 PMC9562128

[104] Gold L. Aptamer-based multiplexed proteomic technology for biomarker discovery. PloS one 5, e15004 (2010). 21165148 10.1371/journal.pone.0015004 PMC3000457

[105] Yang C. Genomic atlas of the proteome from brain, CSF and plasma prioritizes proteins implicated in neurological disorders. Nature neuroscience 24, 1302–1312 (2021). 34239129 10.1038/s41593-021-00886-6 PMC8521603

[106] Team, R. C. R core team (2014). R: A language and environment for statistical computing. R Foundation for Statistical Computing, Vienna, Austria. URL http://www.R-project.org (2014).

[107] Leek J. T., Johnson W. E., Parker H. S., Jaffe A. E. & Storey J. D. The sva package for removing batch effects and other unwanted variation in high-throughput experiments. Bioinformatics (Oxford, England) 28, 882–883 (2012). 22257669 10.1093/bioinformatics/bts034 PMC3307112

[108] Lun A. T. L., McCarthy D. J. & Marioni J. C. A step-by-step workflow for low-level analysis of single-cell RNA-seq data with Bioconductor. F1000Research 5, 2122 (2016). 27909575 10.12688/f1000research.9501.1 PMC5112579

[109] Stouffer S. A., Suchman E. A., Devinney L. C., Star S. A. & Williams R. M. Jr. The American soldier: Adjustment during army life. (Studies in social psychology in World War II), Vol. 1. The American soldier: Adjustment during army life. (Studies in social psychology in World War II), Vol. 1 (Princeton Univ. Press, 1949).

[110] Won S., Morris N., Lu Q. & Elston R. C. Choosing an optimal method to combine P-values. Statistics in medicine 28, 1537–1553 (2009). 19266501 10.1002/sim.3569 PMC2771157

[111] Kuhn M. Building Predictive Models in R Using the caret Package. Journal of Statistical Software 28, 1–26 (2008).27774042

[112] Marek K. The Parkinson’s progression markers initiative (PPMI) - establishing a PD biomarker cohort. Annals of clinical and translational neurology 5, 1460–1477 (2018). 30564614 10.1002/acn3.644 PMC6292383

[113] Robin X. pROC: an open-source package for R and S+ to analyze and compare ROC curves. BMC Bioinformatics 12, 77 (2011). 21414208 10.1186/1471-2105-12-77 PMC3068975

[114] Ruopp M. D., Perkins N. J., Whitcomb B. W. & Schisterman E. F. Youden Index and optimal cut-point estimated from observations affected by a lower limit of detection. Biometrical journal. Biometrische Zeitschrift 50, 419–430 (2008). 18435502 10.1002/bimj.200710415 PMC2515362

[115] Yu G., Wang L.-G., Han Y. & He Q.-Y. clusterProfiler: an R package for comparing biological themes among gene clusters. Omics : a journal of integrative biology 16, 284–287 (2012). 22455463 10.1089/omi.2011.0118 PMC3339379

[116] Zickenrott S., Angarica V. E., Upadhyaya B. B. & Del Sol A. Prediction of disease-gene-drug relationships following a differential network analysis. Cell Death Dis (2016) doi:10.1038/cddis.2015.393. PMC4816176 26775695

[117] Csardi G. & Nepusz T. The igraph software package for complex network research. InterJournal Complex Systems (2006).

[118] Kwon Y.-K., Choi S. S. & Cho K.-H. Investigations into the relationship between feedback loops and functional importance of a signal transduction network based on Boolean network modeling. BMC bioinformatics 8, 384 (2007). 17935633 10.1186/1471-2105-8-384 PMC2100072

[119] PLAHTE E., MESTL T. & OMHOLT S. W. FEEDBACK LOOPS, STABILITY AND MULTISTATIONARITY IN DYNAMICAL SYSTEMS. Journal of Biological Systems 03, 409–413 (1995).

[120] Gouzé J.-L. Positive and Negative Circuits in Dynamical Systems. Journal of Biological Systems (2003) doi:10.1142/s0218339098000054.

[121] Wickham H. Ggplot2: Elegant Graphics for Data Analysis. (Springer Publishing Company, Incorporated, 2009).

[122] Shannon P. Cytoscape: A software Environment for integrated models of biomolecular interaction networks. Genome Research (2003) doi:10.1101/gr.1239303. PMC403769 14597658

[123] Hu C. CellMarker 2.0: an updated database of manually curated cell markers in human/mouse and web tools based on scRNA-seq data. Nucleic Acids Res 51, D870–D876 (2023). 36300619 10.1093/nar/gkac947 PMC9825416

[124] Franzén O., Gan L.-M. & Björkegren J. L. M. PanglaoDB: a web server for exploration of mouse and human single-cell RNA sequencing data. Database (Oxford) 2019, (2019). 10.1093/database/baz046 PMC6450036 30951143

[125] Falcon S. & Gentleman R. Hypergeometric Testing Used for Gene Set Enrichment Analysis BT - Bioconductor Case Studies. in (eds. Hahne F., Huber W., Gentleman R. & Falcon S.) 207–220 (Springer New York, 2008). doi:10.1007/978-0-387-77240-0_14.

[126] Goel M. K., Khanna P. & Kishore J. Understanding survival analysis: Kaplan-Meier estimate. International journal of Ayurveda research 1, 274–278 (2010). 21455458 10.4103/0974-7788.76794 PMC3059453

