# Supplementary material for: Multi-cohort cerebrospinal fluid proteomics identifies robust molecular signatures for asymptomatic and symptomatic Alzheimer’s disease
Source: Res Sq. 2024 Feb 16:rs.3.rs-3631708. Preprint. [Version 1] doi: 10.21203/rs.3.rs-3631708/v1 (PMC10896368; doi:10.21203/rs.3.rs-3631708/v1)
Supplement: Supplement 1 [file NIHPPRS3631708V1-supplement-1.pdf]

Supplementary Files

This is a list of supplementary files associated with this preprint. Click to download.

- 2.ExtendedResults.docx
- 4.SupplementaryTables.xlsx
- 3.ExtendedFigures.docx
